# The Use of Multiple Imaging Studies Before Shoulder Stabilization Surgery Is Increasing

**DOI:** 10.1016/j.asmr.2022.01.003

**Published:** 2022-02-13

**Authors:** Madeleine A. Salesky, Alan L. Zhang, C. Benjamin Ma, Brian T. Feeley, Valentina Pedoia, Drew A. Lansdown

**Affiliations:** a School of Medicine, University of California San Francisco, San Francisco, California, USA; b Department of Orthopaedic Surgery, University of California San Francisco, San Francisco, California, USA

## Abstract

**Purpose:**

To determine the incidence of preoperative shoulder imaging, explore the prevalence of obtaining multiple advanced imaging studies, and identify patient characteristics associated with specific imaging studies before anterior versus posterior shoulder stabilization surgery.

**Methods:**

The PearlDiver database was queried for patients who underwent anterior or posterior shoulder stabilization surgery from 2010 to 2019. The incidence of imaging studies within a year of surgery was collected. Patient characteristics were compared between groups using one-way analysis of variance or χ^2^ test.

**Results:**

In total, 10,252 patients underwent anterior shoulder stabilization surgery, and 1,108 patients underwent posterior shoulder stabilization surgery. Imaging use before anterior and posterior shoulder stabilization surgery included plain radiographs (69%, 70%, respectively), magnetic resonance imaging (MRI; 43%, 33%), and computed tomography (CT; 22%, 22%). In total, 1,098 patients (11%) received MRI and CT before anterior stabilization surgery and 85 patients (8%) received MRI and CT before posterior stabilization surgery. Over time, the incidence of obtaining MRI and CT increased before anterior (z = 2.54, *P* = .011) and posterior (z = 2.36, *P* = .018) stabilization surgery.

**Conclusions:**

This study highlights the increasing use of multiple imaging studies before shoulder stabilization surgery over recent years, including plain radiographs, MRI, and CT imaging. In total, 45% of anterior shoulder stabilization patients and 41% of posterior shoulder stabilization patients obtained more than 1 imaging study within a year of surgery, with a recent increase in patients obtaining both MR and CT scans preoperatively.

**Statement of Clinical Relevance:**

The increasing use of multiple preoperative imaging studies observed in this study highlights an opportunity for new imaging technology to streamline and improve the preoperative workup.

Function and stability of the glenohumeral joint relies on the bony anatomy of the glenoid and humeral head as well as the integrity of the labrum, capsule, and rotator cuff tendons and musculature. For recurrent anterior shoulder instability, patients with greater than 20% to 25% bone loss at the glenoid have failure rates upwards of 70% with arthroscopic capsulolabral repair.^1^^,^^2^ Even lower levels of glenoid bone loss may impact biomechanical outcomes, including standardized functional and instability scores after surgery without recurrent dislocation.^3^^,^^4^ There has been a recent increase in the use of bony augmentation procedures in the management of shoulder instability, as well as numerous advancements in arthroscopic technology for managing bony deficiencies in shoulder instability.^5^ These novel technologies and surgical approaches allow surgeons to appropriately tailor treatment decisions when glenoid bone abnormalities are recognized pre-operatively.^6^^,^^7^

Proper preoperative determination of the status of the glenoid, capsulolabral tissue, and rotator cuff is necessary to comprehensively treat shoulder instability. Glenoid bone loss and pathologic wear patterns can be difficult to evaluate with many traditional diagnostic modalities. Previous studies have demonstrated that 3-dimensional (3D) reconstruction of computed tomography (CT) scans allows for the best evaluation of these parameters.^8^, ^9^, ^10^, ^11^ Understanding the status of the labrum, capsule, and rotator cuff, however, is also essential, and these structures are not visualized well with CT. Magnetic resonance imaging (MRI) and magnetic resonance arthrograms (MRA) are commonly used to assess the extent of capsular and labral injury, presence of rotator cuff tears, degree of rotator cuff muscle atrophy, and other soft-tissue pathologies.^12^ There have been multiple reports of novel sequences or processing techniques to allow for 3D reconstructions from MRI, although these protocols are not widely used yet in clinical practice.^9^^,^^13^^,^^14^

Despite the considerable role of imaging studies in preoperative evaluation, variability in clinical practice can result in substantial differences in the cost and quality of care. Before shoulder stabilization surgery, patients may undergo several advanced imaging studies including MRI and CT scans to evaluate soft tissue and bony structures. Obtaining multiple imaging studies before surgery can drastically increase the cost and inconvenience of preoperative workup for patients before shoulder surgery, while at other times may allow for development of a complete and comprehensive preoperative plan.^15^^,^^16^ A better understanding of how imaging studies are used in preoperative workup for shoulder instability is necessary to address these inconsistencies and understand the role that new technologies may have in simplifying the preoperative diagnostic process for these patients.

The purposes of this study were to determine the incidence of preoperative shoulder imaging, explore the prevalence of obtaining multiple advanced imaging studies, and identify patient characteristics associated with specific imaging studies before anterior versus posterior shoulder stabilization surgery. We hypothesized that the use of multiple imaging modalities per patient for preoperative evaluation would show increased frequency over recent years for patients undergoing anterior and posterior shoulder stabilization surgery.

## Methods

### Data Collection

Retrospective data for this study were obtained from a commercially available database, the PearlDiver Bellwether interface (www.pearldiverinc.com; PearlDiver Inc., Fort Wayne, IN). The PearlDiver database includes more than 122 million privately and publicly insured patients including those with private insurance, Medicare, Medicaid, and self-pay. Deidentified patient records can be queried from this database using Current Procedural Terminology (CPT) codes and *International Association of Diseases* (ICD), *Ninth Revision* (-9), and *Tenth Revision* (-10) codes. Data for this study were derived from the Mariner dataset. The database was accessed via a password-protected server maintained by PearlDiver.

Patients who underwent shoulder stabilization surgery from 2010 to 2019 were identified using CPT codes (Table 1). Patients were separated into primary anterior versus posterior shoulder stabilization groups based on the codes listed in Table 1 to determine differences in imaging use between patients with anterior versus posterior instability. Patients younger than 18 years old and patients who underwent both anterior and posterior stabilization surgery during the study period were excluded from analysis.

**Table 1:** Surgical Codes

Shoulder Arthroplasty	Codes
Anterior Shoulder Stabilization Surgery	CPT-23450, CPT-23455, CPT-23460, CPT-23462
Posterior Shoulder Stabilization Surgery	CPT-23465

NOTE. This table depicts the surgical codes used to identify patients who underwent anterior or posterior shoulder stabilization surgery during the study period.

CPT, Current Procedural Terminology.

Patients who obtained preoperative imaging studies within a year of surgery were identified according to ICD-9, ICD-10, and CPT codes for shoulder imaging (Table 2). Given that MRAs are often billed using MRI codes, we were unable to differentiate between MRI and MRA imaging techniques. We limited our analysis to imaging studies obtained before surgery to within a year to select for imaging studies that were most relevant to preoperative planning. Patients who obtained multiple imaging studies within a year of surgery were identified using the codes for each imaging type. The annual incidence of each surgery and imaging study were collected. Patient-level data also were collected for each surgery and imaging group, including age, patient sex, and Charlson Comorbidity Index.

**Table 2:** Diagnostic Imaging Codes

Imaging Studies	
Radiographic Imaging	ICD-9-P-8821, ICD-9-P-8824, ICD-10-P-BP08ZZZ, ICD-10-P-BP091ZZ, ICD-10-P-BP09ZZZ, CPT-73020, CPT-73030, CPT-73040
Magnetic Resonance Imaging	ICD-9-P-8894, ICD-9-P-8897, ICD-10-P-BP38Y0Z, ICD-10-P-BP38YZZ, ICD-10-P-BP38ZZZ, ICD-10-P-BP39Y0Z, ICD-10-P-BP39YZZ, ICD-10-P-BP39ZZZ, ICD-10-P-BP3EYZZ, ICD-10-P-BP3EZZZ, ICD-10-P-BP3FY0Z, ICD-10-P-BP3FYZZ, ICD-10-P-BP3FZZZ, CPT-73221, CPT-73222, CPT-73223, CPT-73225
Computed Tomography Imaging	ICD-9-P-8838, ICD-10-P-BP28ZZZ, ICD-10-P-BP29YZZ, ICD-10-P-BP29ZZZ, ICD-10-P-BP2E1ZZ, ICD-10-P-BP2EZZZ, ICD-10-P-BP2F1ZZ, ICD-10-P-BP2T1ZZ, ICD-10-P-BP2TYZZ, ICD-10-P-BP2TZZZ, ICD-10-P-BP2UYZZ, ICD-10-P-BP2UZZZ, ICD-10-P-BP2V1ZZ, CPT-73200, CPT-73201, CPT-73202, CPT-73206

NOTE. the imaging codes used to identify patients who obtained preoperative imaging studies within 1 year of surgery.

CPT, Current Procedural Terminology, ICD, *International Classification of Diseases*.

### Statistical Analyses

Patient characteristics were compared between patients who obtained different imaging tests before anterior versus posterior shoulder stabilization surgery were compared using one-way analysis of variance for continuous variables or χ^2^ test for categorical variables. Trends in obtaining multiple imaging studies before anterior and posterior shoulder stabilization surgery were determined using a nonparametric test of trends of ranks. Statistical analysis was performed on the PearlDiver server and in Stata 16.1 (StataCorp, College Station, TX). Statistical significance was defined as *P* < .05.

## Results

### Surgery Demographics

A total of 11,360 patients underwent shoulder stabilization surgery, including 10,252 patients who underwent anterior shoulder stabilization surgery and 1,108 patients who underwent posterior shoulder stabilization surgery. The median age of patients who underwent anterior shoulder stabilization surgery was 32 years (first to third interquartile range: 23-47 years). 68% of anterior shoulder stabilization patients were male. The median age of patients who underwent posterior shoulder stabilization surgery was 42 years (interquartile range: 28-59 years). In total, 67% of posterior stabilization surgery patients were male.

### Overall Use of Preoperative Imaging Studies

**Fig 1:**
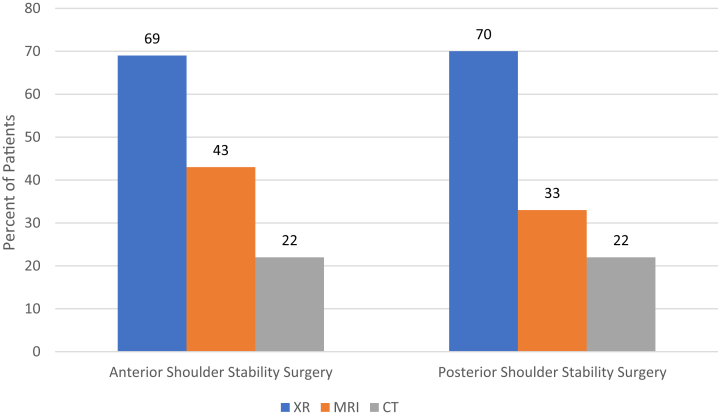
The rates of use of preoperative radiograph (XR), magnetic resonance imaging (MRI), and computed tomography (CT) scans are shown for patients undergoing treatment for shoulder stabilization with anterior or posterior shoulder stability surgery. Individuals who underwent multiple imaging studies are accounted for in their respective imaging modalities, so the percentages for each surgery sum to greater than 100.

Of the 10,252 patients who underwent anterior shoulder stabilization surgery, 7,089 (69%) obtained radiographs (XR), 4,372 (43%) obtained MRI or MRA, and 2,245 (22%) obtained CT imaging within 1 year of surgery. Among the 1,108 patients who underwent posterior shoulder stabilization surgery, 776 (70%) obtained XR, 367 (33%) obtained MRI or MRA, and 244 (22%) obtained CT imaging before surgery. These data are summarized in Figure 1.

### Incidence of Multiple Preoperative Imaging Studies

Before anterior shoulder stabilization surgery, 27% of patients obtained 1 imaging study only, 36% of patients obtained 2 imaging studies, and 9% of patients obtained 3 imaging studies (Table 3). A total of 3,515 anterior shoulder stabilization patients (34%) obtained both XR and MRI, 1,915 patients (19%) obtained XR and CT, and 1,098 patients (11%) obtained CT and MRI imaging before surgery. Before posterior shoulder stabilization surgery, 19% of patients obtained 1 imaging study, 34% of patients obtained 2 imaging studies, and 7% of patients obtained 3 imaging studies (Table 3). Among posterior shoulder instability patients, 300 patients (27%) obtained XR and MRI, 211 patients (19%) obtained XR and CT, and 85 patients (8%) obtained CT and MRI imaging before surgery.

**Table 3:** Incidence of Obtaining Multiple Imaging Studies

	Total	One Imaging Study (%)	Two Imaging Studies, n (%)	Three Imaging Studies, n (%)
Anterior shoulder stability surgery	10,252	2,795 (27%)	3,672 (36%)	952 (9%)
Posterior shoulder stability surgery	1,108	21 (19%)	377 (34%)	73 (7%)

NOTE. This table depicts the proportion of anterior shoulder stability patients and posterior shoulder stability patients who obtained a single imaging study, 2 imaging studies, or 3 imaging studies before surgery.

**Fig 2:**
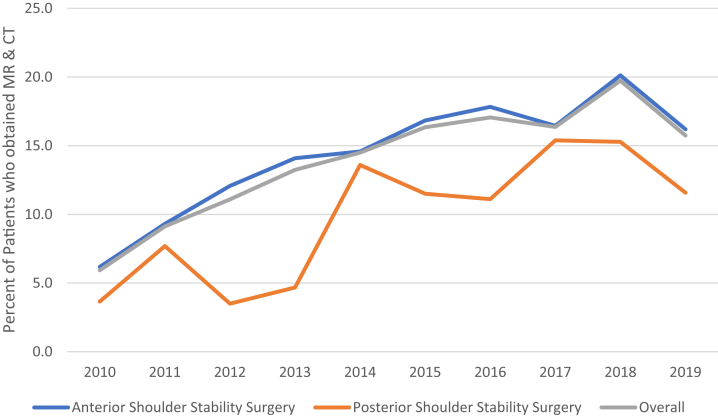
The rate of use of preoperative magnetic resonance (MR) imaging and computed tomography (CT) scans is shown for patients who underwent anterior shoulder stability surgery, patients who underwent posterior shoulder stability surgery, and combined patients who underwent anterior or posterior shoulder stability surgery. The rate of use of both advanced imaging studies has increased significantly for anterior (z = 2.54; *P* = .011), and posterior (z = 2.36; *P* = .018) shoulder stability surgery patients, and patients overall (z = 2.62; *P* = .009).

A total of 1,183 patients obtained both MRI and CT imaging studies before surgery including 1,098 patients who underwent anterior shoulder stabilization surgery (11%) and 85 patients who underwent posterior shoulder stabilization surgery (8%, Fig 2). The percentage of patients who obtained both MR and CT before surgery each year increased significantly between 2010 and 2019 for patients undergoing anterior (z = 2.54, *P* = .011) and posterior (z = 2.36, *P* = .018) stabilization surgery, as well as overall (z = 2.62, *P* = .009*).*

### Comparison of Patient Demographics by Imaging Use

The demographics of patients who underwent different imaging studies before anterior or posterior shoulder stabilization surgery are summarized in Table 4. Among both groups, patients who underwent CT and not MRI before surgery were older than patients who underwent other preoperative imaging studies (Table 4). Among patients who underwent anterior shoulder stabilization, patients who underwent both CT and MRI before surgery were younger than those who underwent other preoperative imaging studies (Table 4). Patients who obtained CT and MRI before anterior stabilization surgery were also more frequently male (80.0%) than other groups. Among posterior instability patients, patients who obtained CT and MRI had more medical comorbidities than patients who obtained other preoperative imaging studies (*P* = .004, Table 4).

**Table 4:** Comparison of Patient Demographics by Imaging Study Use

	Mean Age, y (*P* Value)	Mean CCI (*P* Value)	Patient Sex, % Male (*P* Value)
Anterior shoulder stability surgery			
XR only	37.2 (*P* = .01)	0.65 (*P* = .66)	66.6% (*P* < .001)
MRI only (with or without XR)	35.5 (*P* = .01)	0.63 (*P* = .55)	67.7% (*P* < .001)
CT only (with or without XR)	38.6 (*P* < .001)	0.93 (*P* < .001)	69.5% (*P* < .001)
CT and MRI (with or without XR)	33.5 (*P* < .001)	0.74 (*P* = .001)	80.0% (*P* = .reference)
Posterior shoulder stability surgery			
XR only	44.2 (*P* = .34)	0.88 (*P* = .05)	67.6% (*P* = .90)
MRI only (with or without XR)	39.3 (*P* < .001)	0.82 (*P* = .74)	64.5% (*P* = .53)
CT only (with or without XR)	45.8 (*P* = .17)	0.91 (*P* = .66)	75.5% (*P* = .22)
CT and MRI (with or without XR)	40.1 (*P* = .02)	1.11 (*P* = .004)	68.2% (*P* = reference)

NOTE. This table demonstrates differences in patient age, sex, and between patients who obtained different preoperative imaging workups before anterior or posterior shoulder stabilization surgery.

CCI, Charlson Comorbidity Index; CT, computed tomography; MRI, magnetic resonance imaging; XR, plan radiograph.

## Discussion

We observed that an increasing proportion of patients with shoulder instability is obtaining multiple advanced imaging studies within one year before surgery. Overall, 25% of patients obtained 1 imaging study and 45% of patients obtained 2 or more imaging studies. While the bony and soft-tissue findings on preoperative imaging studies are an essential component of surgical evaluation, the utility of obtaining multiple advanced imaging studies before shoulder surgery is not clear.

Among stabilization surgery patients, MRI was more frequently obtained in conjunction with plain radiographs than CT plus SR in this study. No differences were observed for patients undergoing treatment for anterior or posterior instability. For these patients who obtained MR and XR imaging, there may be suboptimal evaluation of abnormal glenoid wear patterns or subcritical glenoid bone loss.^4^ Incomplete recognition of glenoid bone loss or glenoid wear patterns may negatively influence clinical outcomes, and the lack of detection may in part be due to information acquired in the preoperative evaluation.

Concerning the association between patient demographics and the use of different preoperative imaging techniques, we observed that female patients with anterior shoulder instability were less likely to have both MRI and CT before surgery. This trend was not observed for patients who underwent posterior shoulder stabilization surgery, suggesting a possible disparity in care. Patients who obtained CT and not MRI before both anterior and posterior stabilization surgery were older on average than patients who obtained XR only, MRI only, or CT and MRI before surgery. Previous reports have shown that increasing age and male sex are predictors for glenohumeral bone and cartilage lesions.^17^ This observation in our study potentially reflects this in clinical practice, although this pattern may also lead to underdetection of pathology in these patient groups. Future studies should build off of these findings to investigate whether certain patient groups are more likely to have different preoperative workups and to determine the clinical impact and generalizability of the differences observed in this study.

We did identify a recent increase in multiple imaging studies obtained before shoulder stabilization surgery. A similar trend was noted for both anterior and posterior instability. This observation can likely be attributed to the increased recognition of the contribution of both bony and soft tissue injury management for patients with recurrent instability.^3^^,^^4^ Soft-tissue injuries, such as labral injuries, humeral avulsion of the glenohumeral ligaments, or potential rotator cuff pathology, can be detected best with MRI or MRA.^18^, ^19^, ^20^ Even lower levels of glenoid bone loss and the presence of Hill–Sachs lesions can prompt changes in surgical plan, and these abnormalities are best detected currently with 3D CT reconstructions.^10^ Intra-articular bone and cartilage lesions are observed at similar rates for both anterior and posterior instability patients.^17^^,^^21^ Overall, this increase in imaging use for a subset of both anterior and posterior shoulder instability patients likely emphasizes surgeon recognition of the importance in a comprehensive preoperative workup to determine the appropriate surgical plan.

The observations in this study highlight the potential benefit that complete bony evaluation from MRI alone could have in patients with shoulder instability. Ideally, both soft-tissue and bony abnormalities could be detected on a single imaging study to streamline care and reduce cost for the large proportion of patients who underwent multiple imaging studies in this cohort. In the setting of shoulder instability, 3D CT is the gold standard for evaluating osseous defects like glenoid bone loss.^8^^,^^9^^,^^22^ 3D CT involves acquiring high-resolution thin slices, which are then compiled into a 3D volume-rendered reformat to provide better visualization of the glenoid fossa compared with traditional CT.^8^ However, 3D CT imaging involves ionizing radiation exposure which can have negative health impacts over time, especially for young patients with chronic joint instability. Automated 3D MR has demonstrated similar estimates of bone loss, with differences between 3D MR and CT estimates ranging from 0% to 6%.^9^^,^^14^^,^^23^ Manually segmented 3D MRI also provides accurate measurements of bone loss, which are highly correlated with intraoperative estimates, but manual segmentation is less likely to be clinically feasible, given the time and laboratory resources required.^14^^,^^22^^,^^24^ New imaging methods in addition to 3D MRI and 3D CT, especially specific MRI sequences or advanced processing techniques that would require fewer resources, may be able to identify a broader range of pathologies in the future. More research on the accuracy and clinical impact of 3D MRI and other advanced processing techniques is necessary for patients with shoulder instability. The potential use of 3D MRI alone for comprehensive evaluation of both bone and soft-tissue condition could potentially eliminate the duplication of advanced imaging studies in the 10% of patients who obtained MR and CT in this study. In addition, for the 9% of patients in this study who had only one preoperative imaging study of either CT or MRI, a single study that provides more comprehensive information could provide a simpler preoperative workup.

The incidence of obtaining imaging studies including XR, CT, and MRI was lower in this study compared with previous literature. A multicenter descriptive study by Kraeutler et al.^25^ found that 94% of patients undergoing surgical intervention for shoulder instability had preoperative radiographs, 28% of which demonstrated bony deficiency. CT scans were obtained in 12% of patients and MRI was obtained in 92% of patients.^26^ The decreased incidence of imaging studies in our cohort may reflect differences in the study samples, with the MOON study reflecting the practice of high-volume shoulder surgeons and the PearlDiver database being more representative of general practitioners. We did include all imaging studies within 1 year prior to surgical treatment. For those patients without imaging coded, the decision for surgery may be based on symptoms and physical examination alone. Alternatively, these differences may be due to a difference in patient demographics, insurance coverage, or limitations in how procedures were coded.

### Limitations

There are limitations to this study. Our analysis was limited by the variables that were available in the database. Pertinent patient information such as race and ethnicity, income, symptom severity, duration of symptoms, intraoperative findings, and patient-reported outcome measures was unavailable. The laterality of shoulder surgery was not able to be discerned from CPT codes. We were also unable to comment on differences in obtaining MRI versus MRA imaging.

## Conclusions

This study highlights the increasing use of multiple imaging studies before shoulder stabilization surgery over recent years including XR, MR, and CT imaging. In total, 45% of anterior shoulder stabilization patients and 41% of posterior shoulder stabilization patients obtained more than 1 imaging study within a year of surgery, with a recent increase in patients obtaining both MR and CT scans preoperatively.

## Supplementary Data

ICMJE author disclosure forms
