# Supplementary material for: The Use of Multiple Imaging Studies Before Shoulder Stabilization Surgery Is Increasing
Source: Arthrosc Sports Med Rehabil. 2022 Feb 13;4(3):e919–25. doi: 10.1016/j.asmr.2022.01.003 (PMC9210371; doi:10.1016/j.asmr.2022.01.003)
Supplement: ICMJE author disclosure forms [file mmc1.pdf]

ICMJE Form for Disclosure of Potential Conflicts of Interest

Section 1. Identifying Information

1. Given Name _Madeleine 2. Surname ___Salesky____
3. Are you the corresponding author? Yes ___ No_X__
4. Effective Date: 9/8/2021
5. Manuscript Title: Imaging Utilization Prior to Shoulder Surgery

Section 2. The Work Under Consideration for Publication

Did you or your institution at any time receive payment or services from a third party for any aspect of the submitted work (including but not limited to grants, data monitoring board, study design, manuscript preparation, statistical analysis, etc…)?

Complete each item by typing an X in answer yes or not and completing the information requested if an answer is Yes. If you have more than one relationship, add lines.

1. Grant

_X__No ___Yes, money paid to you ___Yes, money paid to institution* Name of entity___
Comments†___

2. Consulting fee or honorarium

_X__No ___Yes, money paid to you ___Yes, money paid to institution* Name of entity___
Comments†___

3. Support for travel to meetings for the study or other purposes

__X_No ___Yes, money paid to you ___Yes, money paid to institution* Name of entity___
Comments†___

4. Fees for participation in review activities such as data monitoring boards, statistical analysis, end-point committees, and the like

__X_No ___Yes, money paid to you ___Yes, money paid to institution* Name of entity___
Comments†___

5. Payment for writing or reviewing the manuscript

__X_No ___Yes, money paid to you ___Yes, money paid to institution* Name of entity___
Comments†___

6. Provision of writing assistance, medicines, equipment, or administrative support

_X__No ___Yes, money paid to you ___Yes, money paid to institution* Name of entity___
Comments†___

7. Other

_X__No ___Yes, money paid to you ___Yes, money paid to institution* Name of entity___
Comments†___

* This means money that your institution received for your efforts on this study.
† Use this section to provide any needed explanation.

Section 3. Relevant financial activities outside the submitted work

1. Board membership

__X_No ___Yes, money paid to you ___Yes, money paid to institution* Name of entity___ Comments___

2. Consultancy

__X_No ___Yes, money paid to you ___Yes, money paid to institution* Name of entity___ Comments___

3. Employment

__X_No ___Yes, money paid to you ___Yes, money paid to institution* Name of entity___ Comments___

4. Expert testimony

__X_No ___Yes, money paid to you ___Yes, money paid to institution* Name of entity___ Comments___

5. Grants/grants pending

_X__No ___Yes, money paid to you ___Yes, money paid to institution* Name of entity___ Comments___

6. Payment for lectures including service on speakers bureaus

_X__No ___Yes, money paid to you ___Yes, money paid to institution* Name of entity___ Comments___

7. Payment for manuscript preparation

_X__No ___Yes, money paid to you ___Yes, money paid to institution* Name of entity___ Comments___

8. Patents (planned, pending or issued)

_X__No ___Yes, money paid to you ___Yes, money paid to institution* Name of entity___ Comments___

9. Royalties

_X__No ___Yes, money paid to you ___Yes, money paid to institution* Name of entity___ Comments___

10. Payment for development of educational presentations

__X_No ___Yes, money paid to you ___Yes, money paid to institution* Name of entity___ Comments___

11. Stock/stock options

__X_No ___Yes, money paid to you ___Yes, money paid to institution* Name of entity___ Comments___

12. Travel/accommodations/ meeting expenses unrelated to activities listed**

_X__No ___Yes, money paid to you ___Yes, money paid to institution* Name of entity___ Comments___

13. Other (err on the side of full disclosure)

__X_No ___Yes, money paid to you ___Yes, money paid to institution* Name of entity___ Comments___

** For example, if you report a consultancy above there is no need to report travel related to that consultancy on this line.

Section 4. Other relationships

Are there other relationships or activities that readers could perceive to have influenced, or that give the appearance of potentially influencing, what you wrote in the submitted work?

__X_No other relationships/conditions/circumstances that present a potential conflict of interest

___Yes, the following relationships/conditions/circumstances are present (explain below):

>>>>>>>>>>><<<<<<<<<<

The International Committee of Medical Journal Editors

ICMJE Form for Disclosure of Potential Conflicts of Interest

Section 1. Identifying Information

1. Given Name _Valentina__________ 2. Surname ___Pedoia______________________
3. Are you the corresponding author? Yes ___ No_X_
4. Effective Date __8 September 2021_____________________
5. Manuscript Title Imaging Utilization Prior to Shoulder Surgery

Section 2. The Work Under Consideration for Publication

Did you or your institution at any time receive payment or services from a third party for any aspect of the submitted work (including but not limited to grants, data monitoring board, study design, manuscript preparation, statistical analysis, etc…)? No

Complete each item by typing an X in answer yes or not and completing the information requested if an answer is Yes. If you have more than one relationship, add lines.

1. Grant

_X__No ___Yes, money paid to you ___Yes, money paid to institution* Name of entity___
Comments†___

2. Consulting fee or honorarium

X___No ___Yes, money paid to you ___Yes, money paid to institution* Name of entity___
Comments†___

3. Support for travel to meetings for the study or other purposes

_X__No ___Yes, money paid to you ___Yes, money paid to institution* Name of entity___
Comments†___

4. Fees for participation in review activities such as data monitoring boards, statistical analysis, end-point committees, and the like

__X_No ___Yes, money paid to you ___Yes, money paid to institution* Name of entity___
Comments†___

5. Payment for writing or reviewing the manuscript

_X__No ___Yes, money paid to you ___Yes, money paid to institution* Name of entity___
Comments†___

6. Provision of writing assistance, medicines, equipment, or administrative support

__X_No ___Yes, money paid to you ___Yes, money paid to institution* Name of entity___
Comments†___

7. Other

__X_No ___Yes, money paid to you ___Yes, money paid to institution* Name of entity___
Comments†___

* This means money that your institution received for your efforts on this study.
† Use this section to provide any needed explanation.

Section 3. Relevant financial activities outside the submitted work

1. Board membership

__X_No ___Yes, money paid to you ___Yes, money paid to institution* Name of entity___ Comments___

2. Consultancy

__X_No ___Yes, money paid to you ___Yes, money paid to institution* Name of entity___ Comments___

3. Employment

__X_No ___Yes, money paid to you ___Yes, money paid to institution* Name of entity___ Comments___

4. Expert testimony

__X_No ___Yes, money paid to you ___Yes, money paid to institution* Name of entity___ Comments___

5. Grants/grants pending

__X_No ___Yes, money paid to you ___Yes, money paid to institution* Name of entity___ Comments___

6. Payment for lectures including service on speakers bureaus

__X_No ___Yes, money paid to you ___Yes, money paid to institution* Name of entity___ Comments___

7. Payment for manuscript preparation

__X_No ___Yes, money paid to you ___Yes, money paid to institution* Name of entity___ Comments___

8. Patents (planned, pending or issued)

__X_No ___Yes, money paid to you ___Yes, money paid to institution* Name of entity___ Comments___

9. Royalties

__X_No ___Yes, money paid to you ___Yes, money paid to institution* Name of entity___ Comments___

10. Payment for development of educational presentations

__X_No ___Yes, money paid to you ___Yes, money paid to institution* Name of entity___ Comments___

11. Stock/stock options

__X_No ___Yes, money paid to you ___Yes, money paid to institution* Name of entity___ Comments___

12. Travel/accommodations/ meeting expenses unrelated to activities listed**

__X_No ___Yes, money paid to you ___Yes, money paid to institution* Name of entity___ Comments___

13. Other (err on the side of full disclosure)

__X_No ___Yes, money paid to you ___Yes, money paid to institution* Name of entity___ Comments___

** For example, if you report a consultancy above there is no need to report travel related to that consultancy on this line.

Section 4. Other relationships

Are there other relationships or activities that readers could perceive to have influenced, or that give the appearance of potentially influencing, what you wrote in the submitted work?

__X_No other relationships/conditions/circumstances that present a potential conflict of interest

___Yes, the following relationships/conditions/circumstances are present (explain below):

>>>>>>>>>>><<<<<<<<<<<

The International Committee of Medical Journal Editors

ICMJE Form for Disclosure of Potential Conflicts of Interest

Section 1. Identifying Information

1. Given Name ___Alan____ 2. Surname __Zhang_____________
3. Are you the corresponding author? Yes ___ No_X__
4. Effective Date: 9/8/2021
5. Manuscript Title: Imaging Utilization Prior to Shoulder Surgery

Section 2. The Work Under Consideration for Publication

Did you or your institution at any time receive payment or services from a third party for any aspect of the submitted work (including but not limited to grants, data monitoring board, study design, manuscript preparation, statistical analysis, etc…)?

Complete each item by typing an X in answer yes or not and completing the information requested if an answer is Yes. If you have more than one relationship, add lines.

1. Grant

__X_No ___Yes, money paid to you ___Yes, money paid to institution* Name of entity___
Comments† ___

2. Consulting fee or honorarium

__X_No ___Yes, money paid to you ___Yes, money paid to institution* Name of entity___
Comments† ___

3. Support for travel to meetings for the study or other purposes

_X__No ___Yes, money paid to you ___Yes, money paid to institution* Name of entity___
Comments† ___

4. Fees for participation in review activities such as data monitoring boards, statistical analysis, end-point committees, and the like

_X__No ___Yes, money paid to you ___Yes, money paid to institution* Name of entity___
Comments† ___

5. Payment for writing or reviewing the manuscript

_X__No ___Yes, money paid to you ___Yes, money paid to institution* Name of entity___
Comments† ___

6. Provision of writing assistance, medicines, equipment, or administrative support

__X_No ___Yes, money paid to you ___Yes, money paid to institution* Name of entity___
Comments† ___

7. Other

__X_No ___Yes, money paid to you ___Yes, money paid to institution* Name of entity___
Comments† ___

* This means money that your institution received for your efforts on this study.
† Use this section to provide any needed explanation.

Section 3. Relevant financial activities outside the submitted work

1. Board membership

__X_No ___Yes, money paid to you ___Yes, money paid to institution* Name of entity___ Comments___

2. Consultancy

___No __X_Yes, money paid to you ___Yes, money paid to institution* Name of entity: Stryker, Depuy Mitek Comments___

3. Employment

_X__No ___Yes, money paid to you ___Yes, money paid to institution* Name of entity___ Comments___

4. Expert testimony

__X_No ___Yes, money paid to you ___Yes, money paid to institution* Name of entity___ Comments___

5. Grants/grants pending

_X__No ___Yes, money paid to you ___Yes, money paid to institution* Name of entity___ Comments___

6. Payment for lectures including service on speakers bureaus

__X_No ___Yes, money paid to you ___Yes, money paid to institution* Name of entity___ Comments___

7. Payment for manuscript preparation

_X__No ___Yes, money paid to you ___Yes, money paid to institution* Name of entity___ Comments___

8. Patents (planned, pending or issued)

_X__No ___Yes, money paid to you ___Yes, money paid to institution* Name of entity___ Comments___

9. Royalties

_X__No ___Yes, money paid to you ___Yes, money paid to institution* Name of entity___ Comments___

10. Payment for development of educational presentations

__X_No ___Yes, money paid to you ___Yes, money paid to institution* Name of entity___ Comments___

11. Stock/stock options

__X_No ___Yes, money paid to you ___Yes, money paid to institution* Name of entity___ Comments___

12. Travel/accommodations/ meeting expenses unrelated to activities listed**

__X_No ___Yes, money paid to you ___Yes, money paid to institution* Name of entity___ Comments___

13. Other (err on the side of full disclosure)

__X_No ___Yes, money paid to you ___Yes, money paid to institution* Name of entity___ Comments___

** For example, if you report a consultancy above there is no need to report travel related to that consultancy on this line.

Section 4. Other relationships

Are there other relationships or activities that readers could perceive to have influenced, or that give the appearance of potentially influencing, what you wrote in the submitted work?

__X_No other relationships/conditions/circumstances that present a potential conflict of interest

___Yes, the following relationships/conditions/circumstances are present (explain below):

>>>>>>>>>>><<<<<<<<<<<

The International Committee of Medical Journal Editors

ICMJE Form for Disclosure of Potential Conflicts of Interest

Section 1. Identifying Information

1. Given Name ___Brian__________ 2. Surname ___Feeley_____________
3. Are you the corresponding author? Yes ___ No__x_
4. Effective Date __9/8/2021_______________
5. Manuscript Title _________ Imaging Utilization Prior to Shoulder Surgery _____________________

Section 2. The Work Under Consideration for Publication

Did you or your institution at any time receive payment or services from a third party for any aspect of the submitted work (including but not limited to grants, data monitoring board, study design, manuscript preparation, statistical analysis, etc…)?

Complete each item by typing an X in answer yes or not and completing the information requested if an answer is Yes. If you have more than one relationship, add lines.

1. Grant

__x_No ___Yes, money paid to you ___Yes, money paid to institution* Name of entity___
Comments†___

2. Consulting fee or honorarium

__x_No ___Yes, money paid to you ___Yes, money paid to institution* Name of entity___
Comments†___

3. Support for travel to meetings for the study or other purposes

_x__No ___Yes, money paid to you ___Yes, money paid to institution* Name of entity___
Comments†___

4. Fees for participation in review activities such as data monitoring boards, statistical analysis, end-point committees, and the like

_x__No ___Yes, money paid to you ___Yes, money paid to institution* Name of entity___
Comments†___

5. Payment for writing or reviewing the manuscript

_x__No ___Yes, money paid to you ___Yes, money paid to institution* Name of entity___
Comments†___

6. Provision of writing assistance, medicines, equipment, or administrative support

_x__No ___Yes, money paid to you ___Yes, money paid to institution* Name of entity___
Comments†___

7. Other

_x__No ___Yes, money paid to you ___Yes, money paid to institution* Name of entity___
Comments†___

* This means money that your institution received for your efforts on this study.
† Use this section to provide any needed explanation.

Section 3. Relevant financial activities outside the submitted work

1. Board membership

__x_No ___Yes, money paid to you ___Yes, money paid to institution* Name of entity___ Comments___

2. Consultancy

__x_No ___Yes, money paid to you ___Yes, money paid to institution* Name of entity___ Comments___

3. Employment

_x__No ___Yes, money paid to you ___Yes, money paid to institution* Name of entity___ Comments___

4. Expert testimony

_x__No ___Yes, money paid to you ___Yes, money paid to institution* Name of entity___ Comments___

5. Grants/grants pending

___No ___Yes, money paid to you _x__Yes, money paid to institution* Name of entity__NIH, OREF, Orthofix_ Comments___

6. Payment for lectures including service on speakers bureaus

__x_No ___Yes, money paid to you ___Yes, money paid to institution* Name of entity___ Comments___

7. Payment for manuscript preparation

_x__No ___Yes, money paid to you ___Yes, money paid to institution* Name of entity___ Comments___

8. Patents (planned, pending or issued)

__x_No ___Yes, money paid to you ___Yes, money paid to institution* Name of entity___ Comments___

9. Royalties

__x_No ___Yes, money paid to you ___Yes, money paid to institution* Name of entity___ Comments___

10. Payment for development of educational presentations

__x_No ___Yes, money paid to you ___Yes, money paid to institution* Name of entity___ Comments___

11. Stock/stock options

___No __x_Yes, money paid to you ___Yes, money paid to institution* Name of entity_Bioniks, Inc: Kaliber Ai__ Comments___

12. Travel/accommodations/ meeting expenses unrelated to activities listed**

_x__No ___Yes, money paid to you ___Yes, money paid to institution* Name of entity___ Comments___

13. Other (err on the side of full disclosure)

_x__No ___Yes, money paid to you ___Yes, money paid to institution* Name of entity___ Comments___

** For example, if you report a consultancy above there is no need to report travel related to that consultancy on this line.

Section 4. Other relationships

Are there other relationships or activities that readers could perceive to have influenced, or that give the appearance of potentially influencing, what you wrote in the submitted work?

_x__No other relationships/conditions/circumstances that present a potential conflict of interest

___Yes, the following relationships/conditions/circumstances are present (explain below):

>>>>>>>>>>><<<<<<<<<<<

The International Committee of Medical Journal Editors

ICMJE Form for Disclosure of Potential Conflicts of Interest

Section 1. Identifying Information

1. Given Name ___C. Benjamin _____ 2. Surname ____Ma____________________
3. Are you the corresponding author? Yes ___ No_X__
4. Effective Date: September 8th 2021
5. Manuscript Title: Imaging Utilization Prior to Shoulder Surgery

Section 2. The Work Under Consideration for Publication

Did you or your institution at any time receive payment or services from a third party for any aspect of the submitted work (including but not limited to grants, data monitoring board, study design, manuscript preparation, statistical analysis, etc…)?

Complete each item by typing an X in answer yes or not and completing the information requested if an answer is Yes. If you have more than one relationship, add lines.

1. Grant

_X__No ___Yes, money paid to you ___Yes, money paid to institution* Name of entity___
Comments†___

2. Consulting fee or honorarium

_X__No ___Yes, money paid to you ___Yes, money paid to institution* Name of entity___
Comments†___

3. Support for travel to meetings for the study or other purposes

__X_No ___Yes, money paid to you ___Yes, money paid to institution* Name of entity___
Comments†___

4. Fees for participation in review activities such as data monitoring boards, statistical analysis, end-point committees, and the like

_X__No ___Yes, money paid to you ___Yes, money paid to institution* Name of entity___
Comments†___

5. Payment for writing or reviewing the manuscript

_X__No ___Yes, money paid to you ___Yes, money paid to institution* Name of entity___
Comments†___

6. Provision of writing assistance, medicines, equipment, or administrative support

_X__No ___Yes, money paid to you ___Yes, money paid to institution* Name of entity___
Comments†___

7. Other

_X__No ___Yes, money paid to you ___Yes, money paid to institution* Name of entity___
Comments†___

* This means money that your institution received for your efforts on this study.
† Use this section to provide any needed explanation.

Section 3. Relevant financial activities outside the submitted work

1. Board membership

___No ___Yes, money paid to you ___Yes, money paid to institution* Name of entity___ Comments___

2. Consultancy

___No _X__Yes, money paid to you _X__Yes, money paid to institution* Name of entity__Stryker, Conmed, Tornier_ Comments___

3. Employment

___No ___Yes, money paid to you ___Yes, money paid to institution* Name of entity___ Comments___

4. Expert testimony

___No ___Yes, money paid to you ___Yes, money paid to institution* Name of entity___ Comments___

5. Grants/grants pending

___No __X_Yes, money paid to you ___Yes, money paid to institution* Name of entity_Aseculap, Samumed, Zimmer, Conmed__ Comments___

6. Payment for lectures including service on speakers bureaus

_X__No ___Yes, money paid to you ___Yes, money paid to institution* Name of entity___ Comments___

7. Payment for manuscript preparation

_X__No ___Yes, money paid to you ___Yes, money paid to institution* Name of entity___ Comments___

8. Patents (planned, pending or issued)

_X__No ___Yes, money paid to you ___Yes, money paid to institution* Name of entity___ Comments___

9. Royalties

___No _X__Yes, money paid to you ___Yes, money paid to institution* Name of entity_Conmed__ Comments___

10. Payment for development of educational presentations

_X__No ___Yes, money paid to you ___Yes, money paid to institution* Name of entity___ Comments___

11. Stock/stock options

_X__No ___Yes, money paid to you ___Yes, money paid to institution* Name of entity___ Comments___

12. Travel/accommodations/ meeting expenses unrelated to activities listed**

_X__No ___Yes, money paid to you ___Yes, money paid to institution* Name of entity___ Comments___

13. Other (err on the side of full disclosure)

_X__No ___Yes, money paid to you ___Yes, money paid to institution* Name of entity___ Comments___

** For example, if you report a consultancy above there is no need to report travel related to that consultancy on this line.

Section 4. Other relationships

Are there other relationships or activities that readers could perceive to have influenced, or that give the appearance of potentially influencing, what you wrote in the submitted work?

__X_No other relationships/conditions/circumstances that present a potential conflict of interest

___Yes, the following relationships/conditions/circumstances are present (explain below):

>>>>>>>>>>><<<<<<<<<<<

The International Committee of Medical Journal Editors

ICMJE Form for Disclosure of Potential Conflicts of Interest

Section 1. Identifying Information

1. Given Name _Drew___________________ 2. Surname ___Lansdown_________________
3. Are you the corresponding author? Yes __X_ No___
4. Effective Date: 9/8/2021
5. Manuscript Title: Imaging Utilization Prior to Shoulder Surgery

Section 2. The Work Under Consideration for Publication

Did you or your institution at any time receive payment or services from a third party for any aspect of the submitted work (including but not limited to grants, data monitoring board, study design, manuscript preparation, statistical analysis, etc…)?

Complete each item by typing an X in answer yes or not and completing the information requested if an answer is Yes. If you have more than one relationship, add lines.

1. Grant

_x__No ___Yes, money paid to you ___Yes, money paid to institution* Name of entity___
Comments†___

2. Consulting fee or honorarium

_x__No ___Yes, money paid to you ___Yes, money paid to institution* Name of entity___
Comments†___

3. Support for travel to meetings for the study or other purposes

_x__No ___Yes, money paid to you ___Yes, money paid to institution* Name of entity___
Comments†___

4. Fees for participation in review activities such as data monitoring boards, statistical analysis, end-point committees, and the like

__x_No ___Yes, money paid to you ___Yes, money paid to institution* Name of entity___
Comments†___

5. Payment for writing or reviewing the manuscript

_x__No ___Yes, money paid to you ___Yes, money paid to institution* Name of entity___
Comments†___

6. Provision of writing assistance, medicines, equipment, or administrative support

_x__No ___Yes, money paid to you ___Yes, money paid to institution* Name of entity___
Comments†___

7. Other

_x__No ___Yes, money paid to you ___Yes, money paid to institution* Name of entity___
Comments†___

* This means money that your institution received for your efforts on this study.
† Use this section to provide any needed explanation.

Section 3. Relevant financial activities outside the submitted work

1. Board membership

_x__No ___Yes, money paid to you ___Yes, money paid to institution* Name of entity___ Comments___

2. Consultancy

_x__No ___Yes, money paid to you ___Yes, money paid to institution* Name of entity___ Comments___

3. Employment

_x__No ___Yes, money paid to you ___Yes, money paid to institution* Name of entity___ Comments___

4. Expert testimony

_x__No ___Yes, money paid to you ___Yes, money paid to institution* Name of entity___ Comments___

5. Grants/grants pending

___No ___Yes, money paid to you _x__Yes, money paid to institution* Name of entity_AANA, AOSSM, Arthritis Foundation__ Comments___AANA – Arthroscopy Research Grant; AOSSM – Young Investigator Award; Arthritis Foundation – research funding

6. Payment for lectures including service on speakers bureaus

_x__No ___Yes, money paid to you ___Yes, money paid to institution* Name of entity___ Comments___

7. Payment for manuscript preparation

_x__No ___Yes, money paid to you ___Yes, money paid to institution* Name of entity___ Comments___

8. Patents (planned, pending or issued)

___No _x__Yes, money paid to you ___Yes, money paid to institution* Name of entity_Patent pending for suture anchor device__ Comments___

9. Royalties

_x__No ___Yes, money paid to you ___Yes, money paid to institution* Name of entity___ Comments___

10. Payment for development of educational presentations

_x__No ___Yes, money paid to you ___Yes, money paid to institution* Name of entity___ Comments___

11. Stock/stock options

_x__No ___Yes, money paid to you ___Yes, money paid to institution* Name of entity___ Comments___

12. Travel/accommodations/ meeting expenses unrelated to activities listed**

x___No ___Yes, money paid to you ___Yes, money paid to institution* Name of entity___ Comments___

13. Other (err on the side of full disclosure)

___No ___Yes, money paid to you _x__Yes, money paid to institution* Name of entity_Arthrex/Evolution Surgical, Stryker__ Comments__Arthrex/Evolution Surgical – educational support; Stryker – educational support_

** For example, if you report a consultancy above there is no need to report travel related to that consultancy on this line.

Section 4. Other relationships

Are there other relationships or activities that readers could perceive to have influenced, or that give the appearance of potentially influencing, what you wrote in the submitted work?

x___No other relationships/conditions/circumstances that present a potential conflict of interest

___Yes, the following relationships/conditions/circumstances are present (explain below):

>>>>>>>>>>><<<<<<<<<<<

The International Committee of Medical Journal Editors
